# Effectiveness and Patient Compliance of a Needle-Free Injection System in Controlled Ovarian Stimulation in IVF Cycles: A Real-World, Investigator-Initiated, Retrospective, Multicenter, Observational Study

**DOI:** 10.7759/cureus.116084

**Published:** 2026-09-10

**Authors:** Manish Banker, Manika Khanna, Baiju P Ahammed, Kamini Patel, K Kunjimoideen

**Affiliations:** 1 Obstetrics and Gynecology, Reproductive Medicine, Banker IVF and Women’s Hospital, Ahmedabad, IND; 2 Obstetrics and Gynecology, Gaudium IVF Centre, New Delhi, IND; 3 Obstetrics and Gynecology, Reproductive Medicine, NCARE IVF Centre, Kannur, IND; 4 Obstetrics and Gynecology, Vaani IVF Centre, Ahmedabad, IND; 5 Obstetrics and Gynecology, IVF, ARMC AEGIS Hospital, Perintalmanna, IND

**Keywords:** controlled ovarian stimulation, gonadotropins, in-vitro fertilization–embryo transfer, needle-free injection system, oocyte retrieval outcomes, pain score, patient compliance

## Abstract

Background

Limited literature exists supporting the use of a needle-free injection system (NFIS) for in vitro fertilization (IVF) cycles, highlighting an unmet need for painless drug delivery to improve patient compliance and reduce treatment dropouts. We evaluated the effectiveness and patient compliance of NFIS for administering gonadotropins for controlled ovarian stimulation (COS) in IVF-embryo transfer (IVF-ET) cycles.

Methods

This real-world study included female patients aged ≥21 years who underwent COS in IVF-ET cycles from December 2025 to February 2026. Patients received either recombinant follicle-stimulating hormone (r-FSH) (Group-1) or r-FSH with highly purified human menopausal gonadotropin (hMG) (Group-2). The endpoints were the number of mature oocytes and total oocytes retrieved, total dose of gonadotropins used, total number of days of stimulation, serum FSH levels (on day 6 of stimulation and on the day of trigger), total number of follicles >17 mm on the day of trigger, and pain score.

Results

Of 43 patients analyzed (Group-1: 25; Group-2: 18), the mean ± SD total number of days of ovarian stimulation was 10.0 ± 1.2, and the total dose of gonadotropins used was 2458.7 ± 617.8 IU. The total number of days of ovarian stimulation was 10.0 ± 1.0 and 10.1 ± 1.5, and the total dose of gonadotropins used was 2203.9 ± 554.2 IU and 2812.5 ± 530.5 IU, for Group-1 and Group-2, respectively. Similarly, serum FSH levels at day 6 of COS were 18.1 ± 4.4 and 27.8 ± 0.0 IU/L, and on the day of ovulation trigger were 15.8 ± 6.0 and 36.0 ± 28.3 IU/L. Total number of follicles >17 mm on the day of trigger was 12.2 ± 6.5 in Group-1 and 4.8 ± 4.1 in Group-2. Total number of oocytes was 13.4 ± 6.2 and 6.1 ± 7.6, and the total number of metaphase II oocytes retrieved was 11.0 ± 5.4 and 4.8 ± 6.3 for Group-1 and Group-2, respectively. The pain score for administering r-FSH via NFIS in both groups was 1.2 ± 1.3, and for hMG via NFIS in Group-2, it was 3.6 ± 2.2.

Conclusion

NFIS was effective for administering gonadotropins for COS in IVF-ET cycles while ensuring patient compliance with minimal pain.

## Introduction

Infertility is a growing global reproductive public health issue that affects both males and females [1,2]. The global prevalence of infertility is about 10-15%, whereas in India, the estimated prevalence ranges from 3.9-16.8% [3]. Given the rising prevalence of infertility coupled with the expanding adoption of assisted reproductive techniques (ART), such as in vitro fertilization-embryo transfer (IVF-ET), addressing the need to overcome treatment-related obstacles.

Though IVF practice has evolved over the years, there is still a high dropout rate (45% to 60%) in IVF treatment, which lowers the success of IVF [4]. In a study, it was found that stress was considered as the most common reason for dropout from IVF treatment [5]. In another study, it was found that the number of injections and the extent of physical pain were among the important reasons for anxiety and stress present in females prior to the IVF procedure [6]. Needle phobia is a universal phenomenon seen in the general population across all age groups [7]. In a previous study, the prevalence rate of needle fear was found to be 20% to 30% in the age group of 20-40 years [8]. The drawbacks associated with the use of hypodermic needles for drug administration have encouraged and motivated scientists to search for alternatives [9].

Needle-free jet injectors (NFJI) were introduced as a substitute for conventional hypodermic needle-based drug delivery [10]. Originally developed in France in the 1860s, these systems deliver drugs by propelling a narrow liquid jet through a finely engineered nozzle, enabling penetration of the skin and underlying tissues [10]. An internal energy source drives a piston to generate high pressure, producing a rapid stream of liquid or powdered medication. By adjusting the velocity of the jet, drugs can be accurately deposited into intradermal, subcutaneous, or intramuscular layers [11]. Various types of needle-free injection systems (NFIS) have been developed and classified based on the type of load (powder injection, liquid injection, depot or projectile injection), based on the working system (spring system, laser-powered, energy-propelled system, gas-propelled system, shock wave), and mechanism of drug delivery (nano patches, sandpaper-assisted delivery, microneedle and iontophoresis enabled) [12].

There is limited literature supporting the use of NFIS in IVF cycles, highlighting an unmet need for painless drug delivery methods to improve patient compliance and reduce treatment dropouts among Indian infertile women undergoing IVF. The current study evaluated the effectiveness and patient compliance of N-FISTM developed by IntegriMedical (approved by the Maharashtra State Food and Drug Administration (FDA), Mumbai, India, for manufacture for sale or distribution in India) utilizing high-pressure spring-driven technology to administer gonadotropins for controlled ovarian stimulation (COS) in IVF cycles [13].

## Materials and methods

Study design

This was an investigator-initiated, real-world, non-comparative, multicenter, retrospective, observational study to evaluate the effectiveness and patient compliance of NFIS for administering gonadotropins during COS in IVF-ET cycles. The records of the patients registered for IVF-ET at five IVF centers across India (Dr. Banker Healthcare Private Ltd., Ahmedabad, India; Vaani IVF Centre, Ahmedabad, India; Gaudium IVF Centre, New Delhi, India; ARMC AEGIS Hospital, Perithalmanna, India; and Ncare IVF, Kannur, India) were assessed for eligibility based on inclusion and exclusion criteria from December 2025 to February 2026.

Eligibility Criteria

Female patients aged ≥21 years, with a normal uterine cavity and who had undergone COS for IVF were included in the study. Patients with a history of ovarian hyperstimulation syndrome (OHSS), hypersensitivity to recombinant follicle-stimulating hormone (r-FSH) or human menopausal gonadotropin (hMG), patients who were prescribed oral ovulogens or recombinant luteinizing hormone (r-LH), and patients with a history of cardiovascular or pulmonary disease, cancer, chemotherapy, or radiotherapy were excluded.

Study Protocol

This study evaluated the effectiveness and patient compliance of N-FISTM (developed by IntegriMedical and marketed by Intas Pharmaceuticals Ltd.) for administering r-FSH 1200IU (Folisurge) and hMG 1200IU (Menotas XP) (both manufactured and marketed by Intas Pharmaceuticals Ltd.) for COS in IVF-ET cycles, as both r-FSH and hMG are commonly used gonadotropins for COS. The device employs a spring-powered, high-pressure mechanism to deliver the drugs without the use of needles. During administration, a high-velocity jet is generated, forcing the drug through a microscopic opening and into the skin, thereby minimizing pain and eliminating needle-associated risks. The NFIS is capable of delivering volumes ranging from 0.1 to 0.5 mL beneath the skin within a fraction of a second. The system delivers medication via subcutaneous and intramuscular routes (Figure 1) [13,14]. While the drug that is administered subcutaneously through a conventional needle forms a bolus-like structure in the hypodermic region, the NFIS delivers the drug in the form of a plume, thereby ensuring that the drug is delivered to a higher surface area; hence increasing the surface area to volume ratio, leading to uniform dispersion and effective absorption [13].

**Figure 1:**
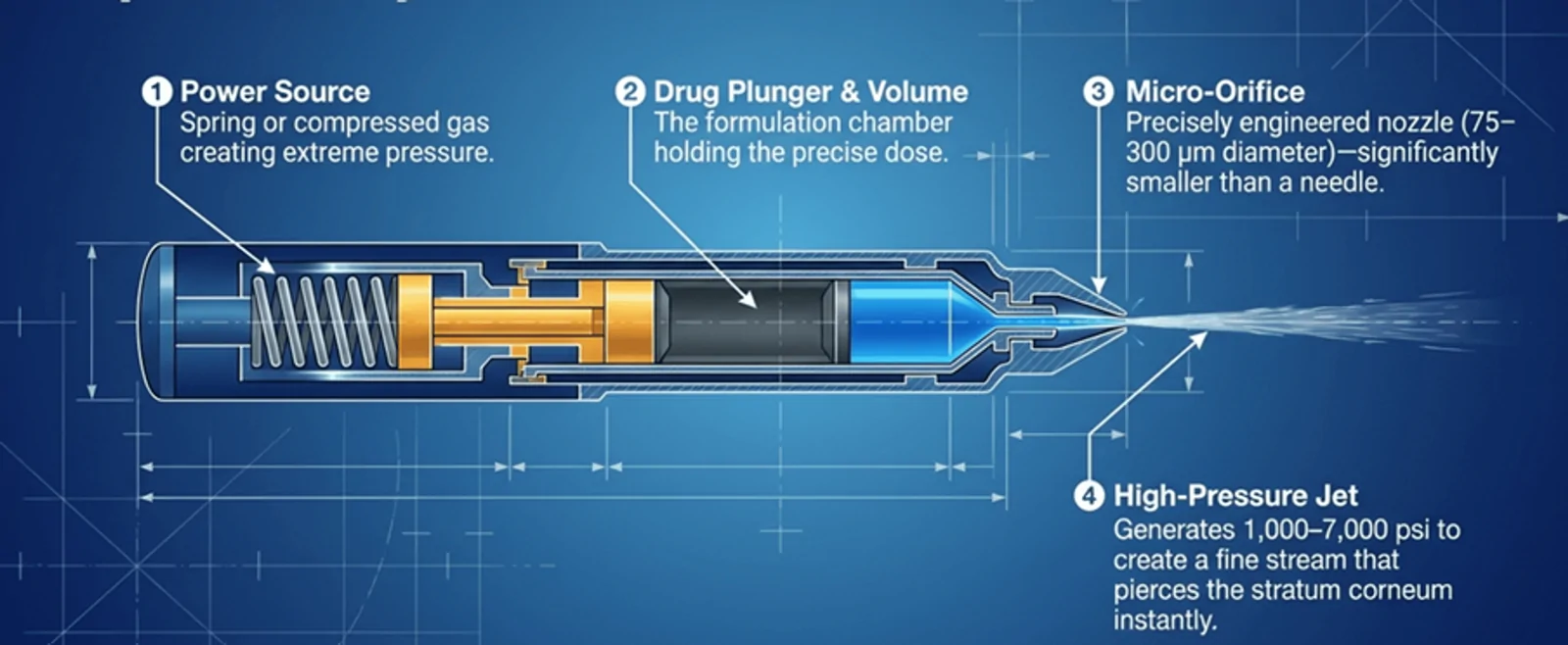
Mechanism of action of a spring-propelled liquid jet injector Image Credits: Manish Banker, using BioRender.com Based on data from: [14]

Interventions and assessments

Though this was not a comparative study, since the patients who visited IVF clinics were of either normal or diminished ovarian reserve and received different COS protocols, subgroup analysis was done on the basis of the type of gonadotropins used.

All patients received either recombinant Follicle Stimulating Hormone (r-FSH: Folisurge 1200IU, manufactured and marketed by Intas Pharmaceuticals Ltd) (Group-1) or r-FSH (Folisurge 1200IU) with highly purified human Menopausal Gonadotropin (hMG: Menotas XP 1200IU, manufactured and marketed by Intas Pharmaceuticals Ltd) (Group-2) by NFIS. hMG was added for patients with diminished ovarian reserve and/or having low serum baseline luteinizing hormone (LH) levels by the clinicians based upon their clinical practice. There was no patient who was administered only hMG via NFIS technology in the said population. The endpoints assessed included number of oocytes retrieved, mature (M2) oocytes retrieved, total dose of gonadotropins, serum FSH levels (at day 6 and trigger), total days of stimulation, total number of follicles developed >17 mm in diameter on the day of trigger, and pain score. Pain score was evaluated using the visual analog scale (VAS) on a scale from 0 to 10, where 0 denotes ‘no pain’ and 10 denotes ‘the most severe pain’ [15].

Data analysis

Descriptive statistics, such as mean ± SD, were used to summarize patients’ baseline and demographic characteristics, dose of gonadotropins administered, duration of COS, serum FSH levels, total count of follicles >17 mm on the day of ovulation trigger, count of oocytes retrieved, and count of M2 oocytes retrieved in both groups. Student's unpaired t-test was used for the statistical calculation. Statistical significance was considered if the P-value was less than 0.05. IBM SPSS Statistics for Windows, Version 20 (Released 2011; IBM Corp., Armonk, New York, USA) was used for statistical analysis.

Ethical considerations

The protocol for the study was reviewed and duly approved by the Independent Ethics Committees (IEC) at all participating sites. The study was conducted according to the ethical principles of the Declaration of Helsinki, ICH-GCP guidelines, ICMR Guidelines for Biomedical Research (2017), and other applicable regulatory requirements. As this was a real-world, retrospective study without patient identifiers and data were retrieved from the patient records, informed consent was waived by the IEC.

## Results

Baseline characteristics

A total of 43 patients (Group-1: 25 patients, Group-2: 18 patients) were included in the analysis. We observed no significant difference (P=0.274) in the mean ± SD age of patients (31.7 ± 5.1 years vs. 33.5 ± 5.3 years for Group-1 vs. Group-2). Similarly, we observed no significant difference in the mean ± SD BMI between the two groups (Table 1). The serum FSH, serum E2, serum TSH, and serum prolactin levels were similar in both groups (Table 1). As the antral follicle count (AFC) and baseline serum LH levels were significantly lower (P=0.002 for both variables) in Group-2, suggesting diminished ovarian reserve, the patients were administered hMG in addition to r-FSH (Table 1).

**Table 1:** Baseline demographic characteristics and investigations *P-value<0.05 Student's unpaired t-test was applied to calculate the P-value. All values are presented as Mean ± SD. # The number of patients was 9, 17, 6, 3, 9, 12, 4, and 12 for BMI, serum AMH, serum FSH, serum LH, serum E2, serum TSH, serum prolactin, and antral follicle count, respectively. AMH, anti-Müllerian hormone; BMI, body mass index; E2, estradiol; FSH, follicle-stimulating hormone; hMG, human menopausal gonadotropin; IU, international unit; LH, luteinizing hormone; NFIS, needle-free injection system; r-FSH, recombinant follicle stimulating hormone; SD, standard deviation; TSH, thyroid stimulating hormone

Characteristics	Patients administered r-FSH alone (n=25) (Group-1)	Patients administered r-FSH with hMG (n=18) (Group-2)	T test (Unpaired)	P-value
Age (years)	31.7 ± 5.1	33.5 ± 5.3	1.108	0.274
BMI (Kg/m^2^)	24.5 ± 3.2	23.7 ± 4.5^#^	0.548	0.587
Serum AMH (ng/mL)	3.8 ± 2.3	2.6 ± 2.7^#^	1.501	0.141
Serum FSH (IU/L)	6.0 ± 4.8	5.2 ± 5.3^#^	0.350	0.729
Serum LH (IU/L)	3.9 ± 1.3	1.2 ± 0.1^#^	3.459	0.002*
Serum E2 (pg/mL)	45.6 ± 12.0	33.3 ± 23.7^#^	2.007	0.053
Serum TSH (mIU/L)	2.3 ± 1.0	2.2 ± 0.9^#^	0.242	0.810
Serum Prolactin (ng/mL)	16.0 ± 7.0	10.6 ± 2.8^#^	1.494	0.147
Antral Follicle Count	16.3 ± 7.5	7.6 ± 6.9^#^	3.379	0.002*

Effectiveness

The mean ± SD total number of days of ovarian stimulation for all patients was 10.0 ± 1.2. The mean ± SD total number of days of ovarian stimulation was 10.0 ± 1.0 and 10.1 ± 1.5 for Group-1 and Group-2, respectively; it was comparable in both groups. Similarly, the mean ± SD total dose of gonadotropins used for all patients was 2458.7 ± 617.8 IU. The mean ± SD total dose of gonadotropins used was significantly higher in Group-2 vs. Group-1 (2812.5 ± 530.5 vs. 2203.9 ± 554.2 IU; P<0.001), respectively, which could be attributed to diminished ovarian reserve in Group-2 patients (Table 2).

**Table 2:** Details of gonadotropin dosage and duration for controlled ovarian stimulation *P-value<0.05 Student's unpaired t-test was applied to calculate the P-value. All values are presented as Mean ± SD. hMG, human menopausal gonadotropin; IU, international unit; NFIS, needle-free injection system; r-FSH, recombinant follicle-stimulating hormone; SD, standard deviation

Parameters	Patients administered r-FSH alone (n=25) (Group-1)	Patients administered r-FSH with hMG (n=18) (Group-2)	T test (Unpaired)	P-value
Total dose (IU) of r-FSH	2203.9 ± 554.2	1383.3 ± 432.2	5.234	<0.001
Total dose (IU) of hMG	-	1423.6 ± 726.6	-	-
Total dose (IU) of gonadotropins	2203.9 ± 554.2	2812.5 ± 530.5	3.616	<0.001*
Total number of days of ovarian stimulation	10.0 ± 1.0	10.1 ± 1.5	0.042	0.967

The mean ± SD serum FSH levels at both day 6 of COS and on the day of ovulation trigger for all patients were 19.0 ± 5.1 IU/L and 17.3 ± 9.6 IU/L for Group-1 and Group-2, respectively. This shows that there was a notable rise in serum FSH levels from baseline in both groups. The serum FSH levels were comparable in both groups at both day 6 of stimulation and the day of ovulation trigger. The total mean ± SD number of follicles >17 mm on the day of trigger for all patients was 9.2 ± 6.7. The mean ± SD total number of follicles >17 mm in diameter on the day of trigger was significantly higher in Group-1 vs. Group-2 (12.2 ± 6.5 vs. 4.8 ± 4.1, P<0.001). The mean ± SD total number of oocytes and M2 oocytes retrieved in all patients was 10.3 ± 7.6 and 8.4 ± 6.5, respectively. The mean ± SD total number of oocytes was significantly higher in Group-1 vs. Group-2 (13.4 ± 6.2 vs. 6.1 ± 7.6, P=0.001). Similarly, the mean ± SD total number of M2 oocytes was also significantly higher in Group-1 vs. Group-2 (11.0 ± 5.4 vs. 4.8 ± 6.3, P=0.001) (Table 3).

**Table 3:** Controlled ovarian stimulation outcomes *P-value<0.05 Student's unpaired t-test was applied to calculate the P-value. All values are presented as Mean ± SD. #n=10, @n=1, $n=2, ^n=17 FSH, follicle-stimulating hormone; hMG, human menopausal gonadotropin; IU, International unit; NFIS, needle-free injection system; r-FSH, recombinant follicle-stimulating hormone; SD, standard deviation

Parameters	Patients administered r-FSH alone (n=25) (Group-1)	Patients administered r-FSH with hMG (n=18) (Group-2)	T test (Unpaired)	P-value
Details of serum FSH levels (IU/L)				
Day 6 of stimulation	18.1 ± 4.4^#^	27.8 ± 0.0^@^	2.100	0.065
On the day of ovulation trigger	15.8 ± 6.0	36.0 ± 28.3^$^	3.381	0.002
Ovarian follicles outcome				
Total number of follicles >17 mm on the day of ovulation trigger	12.2 ± 6.5	4.8 ± 4.1^	4.157	<0.001*
Number of oocytes retrieved	13.4 ± 6.2	6.1 ± 7.6	3.469	0.001*
Number of M2 oocytes retrieved	11.0 ± 5.4	4.8 ± 6.3	3.482	0.001*

Pain score

The mean ± SD pain score for administering r-FSH via NFIS in both groups was 1.2 ± 1.3, and hMG via NFIS in Group-2 was 3.6 ± 2.2 (Table 4).

**Table 4:** Pain scores All values are presented as Mean ± SD. hMG, human menopausal gonadotropin; NFIS, needle-free injection system; r-FSH, recombinant follicle-stimulating hormone; SD, standard deviation

Parameters	N	Pain scores
Administered r-FSH (all patients with or without hMG) by NFIS	43 (Group-1: 25, Group-2: 18)	1.2 ± 1.3
Administered hMG by NFIS	18 (Group-2: 18)	3.6 ± 2.2

Safety

No adverse drug events (ADEs), including serious adverse events (SAEs), such as ovarian hyperstimulation syndrome (OHSS), were observed during the study.

## Discussion

Currently, considerable research efforts are focused on creating technologies that aim at drug administration with improved patient compliance and effectiveness [12]. Conventionally used hypodermic needles for injectable drug delivery present several limitations, including relatively high manufacturing costs of needles, single-use constraints, and reduced patient compliance due to needle-associated anxiety or phobia [16].

NFIs provide a quick and efficient way for drug delivery. Although this technology was initially developed in the mid-nineteenth century, its widespread use became common only after the 1970s, when it began to be used extensively to administer vaccines and antibiotics [17]. The technology promises to make drug administration more effective and less painful, with additional benefits of reducing the possibility of needle stick injuries [12]. Other advantages of NFIS include improved bioavailability compared to invasive drug delivery systems, quicker drug delivery and greater repeatability, reduced needle anxiety, and ease of self-administration [12].

Though the usage of NFIS in the field of IVF has not yet become a common practice, there are very few reports available on the usage of needle-free devices in IVF. In the current context, the present study can contribute to knowledge about the usage of needle-free injector devices for administering gonadotropins in COS in IVF cycles in Indian patients. The NFIS used in the present study is a high-pressure spring-driven jet injector that uses a spring-driven force to deliver the drug into the tissue, where the drug is released into the bloodstream. The NFIS is suitable for both subcutaneous and intramuscular injections [13]. The goal of the current study was to evaluate the effectiveness and patient compliance of NFIS for administering gonadotropins in COS in IVF-ET cycles in the Indian population.

Results of our study showed that the mean ± SD age of all the patients was 32.5 ± 5.2 years, and the BMI was 24.3 ± 3.6 Kg/m^2^ and was comparable in both the groups (for both mean age and mean BMI). In an open-label, single-center, pilot study conducted by Lavery et al. in 20 patients, r-FSH was used for COS in IVF cycles over a period of 3 months (where a J-Tip NFIS was used); the mean ± SD age and BMI of patients were 34.3 ± 4.3 years and 26.2 ± 3.9 Kg/m^2^, respectively [18]. In another prospective randomized trial, conducted by Solnica et al., where a conventional needle was compared with NFIS using CO_2_ gas to generate pressure in 39 patients undergoing COS for IVF, the mean ± SD age in the needle-free CO_2_ injection system group was 35.8 ± 3.8 years, and the traditional needle group was 34.7 ± 3.9 years [19]. Hence, the demographic parameters were comparable in all three studies. Interestingly, in our study, baseline demographics showed that patients in Group-2 had significantly lower AFC and serum LH levels compared to those in Group-1, suggesting diminished ovarian reserve in Group-2 patients.

In our study, the mean ± SD total number of days of COS for all patients was 10.0 ± 1.2 and was comparable in both groups, while in a previous study, the mean ± SD total number of days of COS was 11.0 ± 1.3, i.e., comparable to the results of our study [18]. A previous study compared the COS outcomes between two groups: one receiving gonadotropin via traditional needle and the second via NFIS. It also reported that the mean ± SD total number of injections in the NFIS group and traditional needle group was 9.8 ± 1.6 and 9.5 ± 1.3, respectively [19]. In all these studies, the mean total duration of gonadotropin usage was comparable, and there was no statistically significant difference between the NFIS and traditional needle groups. Hence, it can be suggested that administering gonadotropins via NFIS or conventional needle may have a similar duration of dosage. The mean ±SD total dose of gonadotropins used in our study for all patients was 2458.7 ± 617.8 IU, whereas the same was 1618 ± 427 IU in the previous report [18]. In our study, the mean ± SD total dose of gonadotropins used was significantly higher (P<0.001) in Group-2 (2812.5 ±530.5 IU) than in Group-1 (2203.9 ± 554.2 IU). This could be attributed to the fact that the gonadotropin dose requirement is generally higher for patients with diminished ovarian reserve. Notably, in our study, serum FSH levels at day 6 of COS and on the day of ovulation trigger of all patients were comparable (19.0 vs. 17.3 IU/L).

We observed that the total number of oocytes retrieved in all patients was 10.3, and the total number of oocytes was 13.4 and 6.1 in Group-1 and Group-2, respectively, which could be attributed to small sample size and confounding factors. 

In another study, the mean total number of oocytes collected was 11.8 [18]. The results of our study in Group-1 were comparable to the results of a previous report, which used r-FSH only [18]. Further to note, Solnica et al. reported the median (25th-75th percentile) total number of oocytes was 10 (8-15) in the traditional needle group and 12 (8-15) in the needle-free group, which were comparable to the number of oocytes (both groups) in our study [19].

We also observed a significantly lower (P=0.001) number of M2 oocytes retrieved in patients of Group-2 vs Group-1 (4.8 vs. 11.0). We assume that this could be due to small sample size and confounding factors.

We assessed pain by VAS, a traditional yet reliable tool, which is a widely used psychometric scale offering a simple and effective method for quantifying the intensity or magnitude of subjective pain experiences and attitudes [15]. On a linear scale from 0 to 10, typically, a score lower than 5 denotes lesser pain compared to those having a score higher than 5. In our study, the pain scores were on the lower side for both groups, indicating less pain and hence showed ease of drug administration with better compliance with NFIS. This is consistent with a previous study wherein patients experienced milder pain with NFIS, which was much less than pain experienced by patients with a conventional needle-based injection system [18]. On the contrary, another study did not find any difference in the comfort score and reported that it had a major flaw: the patient enrolment was not restricted to women with higher-than-average fear of needles [19]. Interestingly, in our study, patients administered hMG reported a higher pain score compared to those administered r-FSH. The difference in pain scores between hMG and r-FSH is largely attributed to the inherent differences between urinary-derived and recombinant products.

To the best of our knowledge, this is the first study conducted in an Indian setting to administer gonadotropins for COS in IVF-ET cycles via the NFIS route. This study suggests that the new NFIS technique for administering gonadotropins is effective and ensures patient convenience. NFIS can be the way forward for administering drugs in the setting as IVF. This will help in reducing the patients’ anxiety and psychological stress related to the pain associated with IVF treatment, help in reducing the dropout rates, and enhance the acceptability of IVF therapies.

Due to the retrospective nature, lack of a control arm, and a smaller sample size, the study has some limitations. It is difficult to draw firm conclusions from the study. Prospective studies with a larger study population would be warranted to elucidate the utility of NFIS in the field of IVF-ET.

## Conclusions

Based on the findings of this study, it can be suggested that the N-FISTM used was effective for administering gonadotropins in COS in IVF-ET cycles, in terms of raising serum FSH levels on day 6 of stimulation and on the day of trigger, and the total number of oocytes and M2 oocytes retrieved while ensuring minimal pain to patients. This may help ultimately reduce the dropout rate in IVF therapy because of better compliance. Hence, it can be considered an alternative to conventional injections for administering gonadotropins for COS in IVF cycles. However, larger prospective studies are required to validate these findings.
